# Evolutionary History of the Grey-Faced Sengi, *Rhynchocyon udzungwensis*, from Tanzania: A Molecular and Species Distribution Modelling Approach

**DOI:** 10.1371/journal.pone.0072506

**Published:** 2013-08-27

**Authors:** Lucinda P. Lawson, Cristiano Vernesi, Silvia Ricci, Francesco Rovero

**Affiliations:** 1 Department of Biological Sciences, University of Cincinnati, Cincinnati, Ohio, United States of America; 2 Department of Biodiversity and Molecular Ecology, Centre for Research and Innovation, Michele all’Adige, Italy; 3 Tropical Biodiversity Section, Museo delle Scienze, Trento, Italy; Texas A&M University, United States of America

## Abstract

*Rhynchocyon udzungwensis* is a recently described and poorly understood sengi (giant elephant-shrew) endemic to two small montane forests in Southern Tanzania, and surrounded in lower forests by *R. cirnei reichardi*. In this study, we investigate the molecular genetic relationship between *R. udzungwensis* and *R. c. reichardi*, and the possible role that shifting species distributions in response to climate fluctuations may have played in shaping their evolutionary history. *Rhynchocyon udzungwensis* and *R. c. reichardi* individuals were sampled from five localities for genetic analyses. Three mitochondrial and two nuclear loci were used to construct species trees for delimitation and to determine whether introgression was detectable either from ancient or ongoing hybridization. All species-tree results show *R. udzungwensis* and *R. c. reichardi* as distinct lineages, though mtDNA shows evidence of introgression in some populations. Nuclear loci of each species were monophyletic, implying introgression is exclusively historical. Because we found evidence of introgression, we used distribution data and species distribution modelling for present, glacial, and interglacial climate cycles to predict how shifting species distributions may have facilitated hybridization in some populations. Though interpretations are affected by the limited range of these species, a likely scenario is that the mtDNA introgression found in eastern mid-elevation populations was facilitated by low numbers of *R. udzungwensis* that expanded into lowland heavily occupied *R. c. reichardi* areas during interglacial climate cycles. These results imply that relationships within the genus *Rhynchocyon* may be confounded by porous species boundaries and introgression, even if species are not currently sympatric.

## Introduction

When a new species is described to science, understanding its genetic and morphological distinctness, relationship to congeneric taxa, the permeability of species boundaries, and overall evolutionary history are necessary to place the lineage in the grand scheme of evolution. While many initial investigations document morphological and sometimes molecular relationships to related lineages, considering broader aspects of evolution including non-linear species divergence and interspecific interactions may provide a more accurate estimate of evolutionary history and uniqueness. The prevalence of introgression is non-trivial, as ∼10% of animal species appear to have hybridization and introgression in their evolutionary past [1]. In this study, we investigate the evolutionary history and genetic distinctness of the newly described grey-faced sengi or elephant-shrews (*Rhynchocyon udzungwensis* Rathbun and Rovero 2008) and its relationship to the parapatric chequered sengi (*Rhynchocyon cirnei reichardi* Peters 1847) in the Udzungwa Mountains of Tanzania. Evolutionary relationships within the genus *Rhynchocyon* are poorly understood, and the relationship of the newest member to other East African species and subspecies is of great interest to conservation efforts for preserving unique lineages in the face of small population sizes and human degradation of habitat.

The grey-faced sengi, *R. udzungwensis,* was described in 2008 [2] as the largest member of a poorly known group of African mammals of ancient and intriguing evolutionary history. Sengis are an ancient monophyletic order (Macroscelidea) of 18 extant species, endemic to Africa [3], [4]. Based largely on molecular data, sengis belonging to the super-cohort Afrotheria, which also includes elephants, hyraxes, sea cows (Paenungulata), tenrecs and golden moles (Afrosoricida), and the aardvark (Tubulidentata) [5]–[7]. The sengi order consists of two subfamilies: the forest-dwelling giant sengis (subfamily Rhynchocyoninae, single genus *Rhynchocyon,* species = 4), and the soft-furred, arid adapted sengis (subfamily Macroscelidinae, three genera: *Macroscelides* (species = 2), *Petrodromus* (species = 1), and *Elephantulus* (species = 11)). While a large-scale phylogeny of most sengis has been completed [8], little is known about the taxonomy and phylogenetic relationships of the giant sengis (*Rhynchocyon)* [3], [9]. Corbet & Hanks [10] recognized three *Rhynchocyon* species based on morphological differences and allopatric distribution (four are now recognized with the discovery of *R. udzungwensis*). They also described six subspecies within *R*. *cirnei*, where *R*. *cirnei reichardi* occurs in the Udzungwa Mountains and the highlands of southwestern Tanzania and northern Malawi. This taxonomy, however, is based nearly exclusively on pelage patterns and is debatable in some cases [3], [11]. While sufficient sampling for a robust phylogeny of *Rhynchocyon* is currently beyond our grasp because giant sengis are elusive and sufficient samples are not available, we now have adequate material to investigate the relationship between two parapatric lineages: the newly described *R. udzungwensis* and the more widespread *R. cirnei reichardi.*

**Figure 1:**
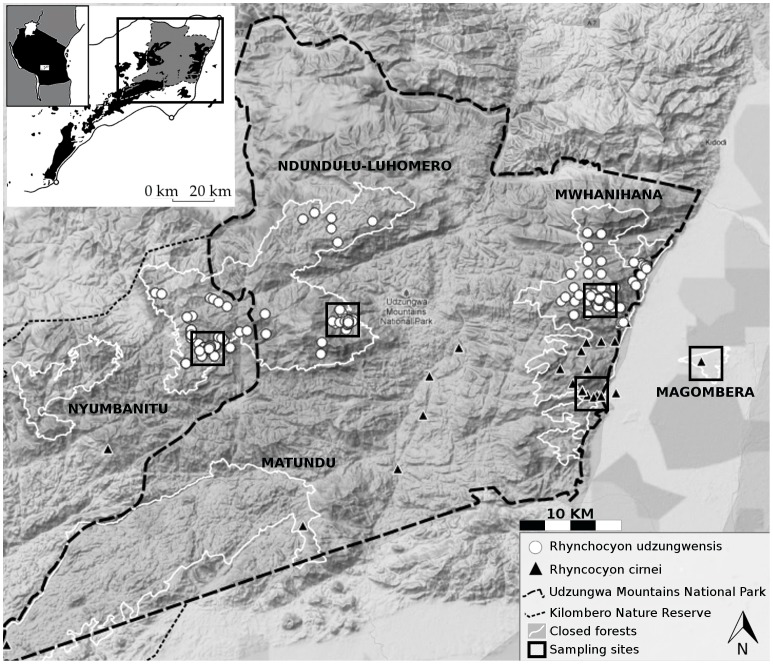
Map of the north-eastern portion of the Udzungwa Mountains, south-central Tanzania with sampling localities. Distribution records of the grey-faced sengi, *Rhynchocyon udzungwensis,* shown as white dots. The occurrence of the checkered sengi, *R. cirnei reichardi,* shown as black triangles. The western portion of the distribution of *R. udzungwensis* is in Ndundulu/Luhomero forest, and the eastern portion is in Mwanihana forest. *Rhynchocyon c. reichardi’s* distribution extends into the Magombera forest on the eastern plain. The five sampling sites for molecular analysis of the two species are also shown. The background layer is a topographic map (dark is lower elevation). The inset shows the location of the Udzungwa Mountains in Tanzania (left) and of the mapped area within the Udzungwa Mountains (right). Insert reprinted from [75] under a CC BY license, with permission from A. Marshall, original copyright 2005.

*Rhynchocyon udzungwensis* has an extremely small range (ca. 390 km^2^) limited to two forests in the northern Udzungwa Mountains in Tanzania: the western Ndundulu-Luhomero forest and the eastern Mwanihana forest, which are separated by a ∼25 km gap of lower elevation and drier woodland [2]. The range of *R. udzungwensis* is nearly encompassed by the range of *R. cirnei reichardi*, and the two taxa present partial overlap in elevation (*R*. *udzungwensis* occurring between 350 and 2300 meters elevation and *R*. *c*. *reichardi* between 290 and 1800 meters in the Udzungwa Mountains [2], [10]). The distributions of the two taxa meet only along the central part of the eastern Mwanihana forest ([12]; Fig. 1), with no indication of physical barriers, abrupt habitat changes, or steep rises. While there is no evidence of intermediate pelage colouration between these two taxa that might suggest hybridization (based on camera-trapping images, sightings, and voucher specimens), the physical proximity of these congeneric lineages, coupled with small population sizes estimated for *R. udzungwensis*, led us to include the relationship with *R. c. reichardi* when investigating *R. udzungwensis’* evolutionary history.

We began this study with a simple investigation of genetic distinction between the two morphological lineages, and then added additional molecular data, species tree analyses, and current and historical species distribution modelling (SDM; see [13] for a review) in order to provide a scenario that could accommodate non-bifurcating molecular data. In this way, we can determine how historical scenarios of contact, isolation, and introgression have shaped evolution for rare and isolated montane fauna, and better understand evolutionary dynamics in a biodiversity hotspot. Species with stable, allopatric distributions are not expected to be prone to hybridization, but species with historic range shifts tracking fluctuating climatic conditions may have experienced temporary sympatry and associated genetic introgression [14]–[17]. While climate-driven range shifts have been documented in a wide variety of settings, the most pronounced shifts are expected in montane species, as climatic similarity may correlate with distant montane areas rather than nearby lowlands [18], [19]. In particular, by using SDM through time, we are able to assess the potential of overlap between sengis during both current and past time periods to accommodate the possibility of low-level historical and/or current introgression [20].

## Materials and Methods

### Ethics Statement

All field procedures involving live animals met the standards for the ethical and humane treatment of animals of the American Society of Mammalogists and the 2000 American Veterinary Medical Association guidelines [21], [22], and all precautions to minimize pain, distress or suffering by trapped animals were taken. Vouchered animals were euthanized using chloroform, as approved by the Guidelines of the American Society of Mammalogists for the use of wild mammals in research [21]. All fieldwork was performed under research/collecting permit number 2008-311-ER-2008-150 to SR and permit number 2010-270-ER-2009-49 to FR, issued by the Tanzania Wildlife Research Institute. Research and collection permits were also issued by the Tanzania Commission for Science and Technology and by National Parks. Materials were exported under Tanzania Wildlife Division/CITES Office export permits #57189 and #58267 to SR.

### Tissue Sampling

We collected tissues for molecular analysis from 31 specimens in Tanzania from 2006–2009: *R. udzungwensis* (n = 22, localities = 3), *R*. *cirnei reichardi* (n = 8, localities = 2), and one outgroup sengi, black-and-rufous sengi (*Rhynchocyon petersi,* n = 1, locality = riparian forest on the north side of the Rufiji river, 150 km east of the Udzungwa Mountains) (Fig. 1). For live animals to be released, tissue collection consisted of a small amount of hair along with a tiny piece of auricle. The ear was then disinfected with betadine and the animal was monitored for a few minutes to ensure no adverse reaction. Dead specimens and specimens destined for voucher had fresh muscle, liver, or kidney tissues preserved in 95% ethanol. Tissue samples for genetic analysis are stored at the Foundation Edmund Mach in Trento, Italy. A complete list of sampled specimens and sampling localities can be found in Table S1.

We trapped sengis with nylon twin snares [2] and double-door live traps (Tomahawk Live Traps, 24×6×6 inch) that were set where sengi were likely to travel. Capture and handling of specimens followed the Animal Care and Use Committee of the American Society of Mammalogists Guidelines. Voucher specimens were collected (*R. udzungwensis*: n = 8 (of which 4 were specimens taken for the description and of these, 3 were given to other museums), *R. cirnei reichardi*: n = 5 (including one given to California Academy of Sciences) and were prepared as study skins and skulls.

### Genetic Analyses

Genomic DNA was extracted with the DNeasy Tissue kit (Qiagen, Valencia, USA) following manufacturer’s instructions. We used PCR to amplify fragments of three mitochondrial loci: NADH dehydrogenase 2 (ND2: Met-1 and Trp-2 [23]), the hypervariable 5′ end of the control region (D-loop: L0-F [24] and E3 [25]) and the ribosomal gene 12S (12S –F & 12S-R, [26]) totalling 1756 bp. For the mtDNA markers we used 10 ng of DNA and PCR conditions were as follows: 94°C for 10 min, followed by 35 cycles of 94°C for 1 min, 55°C for 45 sec, and 72°C for 1 min, with a final extension of 72°C for 10 min. To avoid comparing paralogs, we tested for the presence of numts (for details see Text S1). Starting from 50 ng of extracted DNA, we also amplified portions of two nuclear protein-coding genes, von Willebrand factor (vWF; 1100 bp) and Enamelin (ENAM; 2598 bp), using primers and PCR conditions as in [27]. During both DNA extraction and amplification, blank samples were inserted to check for contamination. All sequence data are deposited in GenBank (Accession Nos. KF202138–KF202289).

Sequencing was performed with the same set of primers used for PCR, using Big Dye terminator cycle sequencing with an ABI 3730×l. Sequences were edited and assembled with Sequencher 4.8 (Gene Codes Corporation). All sequences aligned unambiguously, but we additionally evaluated alignments in MUSCLE (http://www.ebi.ac.uk/Tools/msa/muscle; [28]. Heterozygous genotypes in the nuclear gene data were resolved using PHASE 2.1.1 [29], [30], under the default options of 100 iterations, 1 thinning interval, 100 burn-in iterations, and confidence probability thresholds of 0.90. One nuclear allele from each individual was chosen at random to represent the individual in species-tree analyses.

We concatenated all three mitochondrial loci to form a single mitochondrial dataset for phylogenetic analyses except where specified otherwise. Models of sequence evolution for each locus were selected using the corrected Akaike information criterion (AICc) as implemented in jModelTest [31] (Text S1). We assessed the performance of all Bayesian analyses (convergence and stationarity) with the program Tracer v. 1.5 [32]. Only runs with adequate mixing and ESS above 200 were considered for final analyses. *Rhynchocyon petersi* was included as an outgroup in initial analyses and removed when inclusion failed to alter relationships between *R. c. reichardi* and *R. udzungwensis*.

#### Single gene tree analysis

Each locus was individually analysed using Bayesian inference in MrBayes, [33]. All analyses were run for 200 million Markov chain Monte Carlo (MCMC) generations, sampling every 1000th tree. Half of the resulting trees were discarded as burnin. Maximum clade credibility trees were calculated in TreeAnnotator from Beast v. 1.6.2 [32].

#### Species tree construction

We employed several methods for estimating species trees with different assumptions on ILS (Incomplete Lineage Sorting) and horizontal gene transfer HGT to accommodate the possibility that introgression and HGT may be a substantial part of the evolutionary lineages of at least some populations within these species. Three species tree methods, BEST (Bayesian Estimation of Species Trees: [34], [35], *BEAST **(**Bayesian Evolutionary Analysis Sampling Trees: [36], and BP&P (Bayesian Phylogenetics & Phylogeography: [37], [38] have been shown to accurately estimate phylogenetic (species tree) relationships in spite of gene tree heterogeneity. They assume, however, that ILS is the only cause of gene tree-species tree discordance. To complement these assumptions with a model that is agnostic to the causes of gene tree heterogeneity, we also analysed our dataset in BUCKy (Bayesian Untangling of Concordance Knots, Bayesian concordance analysis: [39]–[41], which can be used to reject ILS as sufficient to explain gene tree discordance. If ILS cannot be rejected as the cause of mismatch between our datasets, then the species-trees produced from *BEAST and BEST should be good estimates of the true species tree without invoking HGT to explain discordance of genetrees. If BUCKy rejects ILS as sufficient to explain genetree discordance, the individual gene trees can be assessed to determine likely transgenic events [42].

BEST v2.3.1 is a hierarchical coalescent model implemented in a two-step MCMC algorithm. The first step estimates the posterior probability distributions of gene trees for each locus in MrBayes, and the second step uses these probabilities to estimate the posterior probability distribution of species trees. In our BEST analysis, we obtained Bayesian posterior probabilities from 100 million MCMC cycles with a sample frequency of 1,000 and a burn-in period of 25 million generations, with a relatively flat prior for θ (α = 3, β = 0.03) as employed in [43], [44]. Models of molecular evolution for each locus were defined from the highest scoring models in jModeltest available in BEST (mtDNA: nst = 2, haploid, Invgamma; ENAM: nst = 2, diploid, Invgamma; vWF: nst = 2, diploid, Equal).

The program *BEAST v. 1.6.2 was used to jointly estimate the posterior distributions of species trees and contained gene trees using coalescent models. The *BEAST analysis was conducted under a strict molecular clock model (no loci violated a strict clock assumption, data not shown) using the SRD06 model of sequence evolution [45] and a random starting tree. As no estimated mutation rates or calibrated phylogenies are available for this clade, relative evolutionary rates were estimated by setting the mean clock rate of mtDNA to 1.0 and allowing all other loci to be estimated in relation to this rate. The final analysis was run for 100 million generations, sampling every 1,000th generation. A maximum clade credibility tree was generated using the program Tree Annotator v.1.6.2 provided in the BEAST package, with a burn-in of 1,000 (10%).

We used the hyperresolved (population level) species tree generated in *BEAST as the user-specified “guide tree” for species tree estimation in the program BPP, v. 2.0. Population size parameters (qs) and the age of the root of the species tree (t0) were assigned gamma priors (α = 2, β = 1000), while all other divergence time parameters are assigned the Dirichlet prior [38]. Each analysis was run twice to confirm consistency between runs. Analyses were run for 150,000 generations, with the first 50,000 discarded as burn-in and a sampling frequency of 5. The species delimitation algorithms 0 and 1 were both run for this dataset (species delimitation: 1 0 5, reported). A variety of algorithms, *a*’s, *e*’s, and *m*’s were explored (algorithm 0, e = 5 & 20. algorithm 1, α = 1 & 2, *m = *0.5, 1, 2) with similar results (Text S1). All five loci (3 mtDNA and 2 nuDNA) were allowed to vary independently in this analysis. Finetune variables were adjusted in the control file for each run so that the acceptance proportions were contained in the interval (0.15, 0.7).

BUCKy v.1.4.0 was used to estimate a primary concordance tree and a population tree from genetrees estimated in MrBayes. Analyses were run for 2 million generations with four different heating chains after a 200,000 generation burn-in. Two extremes of α were explored (α = 0.5, α = 10) with no change to our results. To determine whether HGT appears to play a major role within this region, we compare the Population Tree (PT) and the Primary Concordance Tree (PCT). If ILS is the sole source of gene tree conflict, the two trees should be in tight concordance [46]. If, however the Population Tree and Primary Concordance Tree are in disagreement, this is evidence of hybridization [42].

### Species Distribution Models (SDMs)

#### Occurrence data

Data on the presence of *R. udzungwensis* and *R. cirnei reichardi* were collected from live traps and camera traps [12], museum records, and personal communications (Fig. 1 and Text S1) for a total of 32 and 172 localities for grey-faced and chequered sengi, respectively. To reduce taxonomic misidentification of sengis used in the modelling effort, only data from Tanzania were used to avoid uncertainty of identification and distribution of *R. c. hendersoni* in relation to *R. c. reichardi* [11]. Similarly, samples of *R. c. reichardi* from the western border of Lake Tanganyika were eliminated from our analyses due to uncertainty regarding the affinity with southern Tanzanian samples. Though the recently discovered *Rhynchocyon* in the northern coastal forests of Kenya [47] has pelage colouration and patterning very similar to *R. udzungwensis*, we have not included it in our analyses because it has not been adequately sampled or described.

#### Environmental data

Climate layers were acquired from the WorldClim data set [48]. Three time points representing the recent climate extremes of glacial and interglacial cycles were assessed: current, Last Glacial Maximum (LGM –21 kya, Community Climate System Model: [49], and Last Interglacial (LIG –120 kya: [50]. While these specific time points (21 kya and 120 kya) may not be directly related to range shifts or genetic events concerning these species, by representing well documented dry and wet extremes known to have impacted forest habitat and extent in East Africa, we can approximate the range of distributions experienced by these species. We ran an initial correlation assessment of Bioclim layers at each time point (calculated using ENMtools, v 1.3, [51] to attempt to reduce the number of auto-correlated variables which have the potential of over-fitting models of distribution predictions. As these correlations were not stable between time points or between species, all 19 variables were retained for analyses to avoid under-fitting the model. All layers were clipped to both the extent of Tanzanian boundaries and to a rectangle encompassing the area of species overlap in the Udzungwa Mountains (Latitude: −6° to −9.25°, Longitude: 35° to 37.25°) in ArcGIS (ESRI v.10.1).

#### Species Distribution Models (SDMs)

Species distribution models were created with MaxEnt version 3.3.3 [52]. MaxEnt was chosen because of its ability to make accurate predictions of species distributions on small and spatially limited datasets [53], [54]. All MaxEnt runs were trained on the current climate conditions with a convergence threshold of 0.00001, 1,000 iterations, 25% model testing, logistic output, and the 10% training presence threshold for outputs to create binary models of habitat suitability. Outputs were then projected onto climate models of the LGM and LIG. ENMtools was used to evaluate the similarity of the environmental niche occupied by the two lineages using the Niche Overlap, Niche Identity, and Background functions (200 randomized pseudoreplicates). Background comparisons were completed on using both the distributions envelope of each species (minus the other’s range) from which to generate random points, and the larger shared distribution of the limited area rectangle. The hypothesis of identical climatic niche is rejected when the empirically observed values of identity (D and I statistic: [55], [56] respectively) are significantly lower than the range of pseudoreplicate values [51]. Range overlap through time was calculated in ArcMap.

## Results

### Molecular Analysis

#### Gene trees

**Figure 2:**
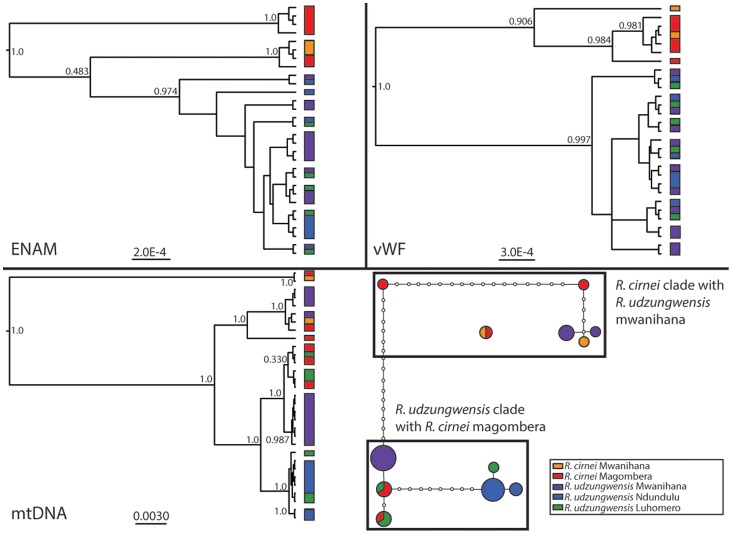
Gene Trees and Haplotype networks. Bayesian Gene trees of two nuclear loci (top: ENAM and vWF) and concatenated mtDNA (bottom left: ND2, *D-loop* region, and the ribosomal gene 12S) constructed in MrBayes. Posterior probabilities displayed for major nodes. Branch lengths proportional to genetic distance. (Bottom Right) Haplotype network of concatenated mtDNA. Each node represents one base pair change.

Nuclear loci were in agreement concerning the monophyly of *R. udzungwensis*. *Rhynchocyon c. reichardi* has strong support of monophyly for vWF, but an uncertain relationship to the *R. udzungwensis* clade in ENAM. Neither species showed intraspecific population structure for nuclear loci (Fig. 2, top). The concatenated mitochondrial loci, however, showed substantial paraphyly of the two species, particularly in regards to *R. c. reichardi* from Magombera and *R. udzungwensis* from Mwanihana forest, which both possessed mtDNA haplotypes from both major clades (Fig. 2, bottom). Dating divergence events between these lineages is currently impossible without a full phylogeny and fossil representation, but future work will help establish the timing of separation of these species and the approximate timing of genetic transfer between species.

#### Species trees

**Figure 3:**
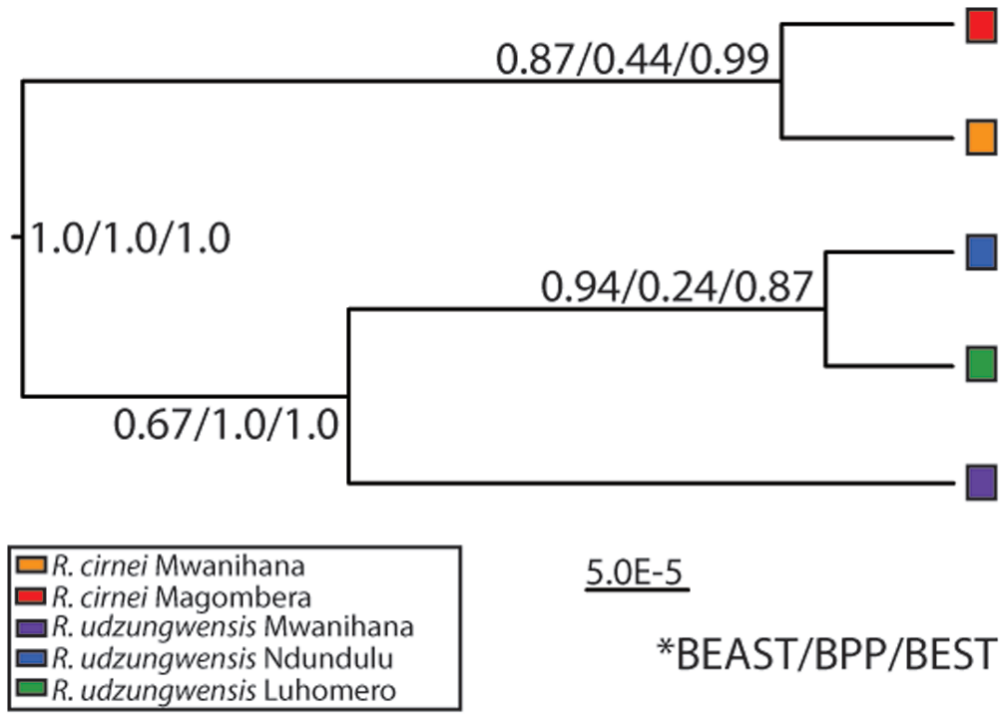
Highest probability species tree selected by all three methods (*BEAST, BPP, and BEST). Posterior probabilities shown for each node. Both species are monophyletic for all species tree reconstructions. Branch lengths proportional to genetic distance.

**Figure 4:**
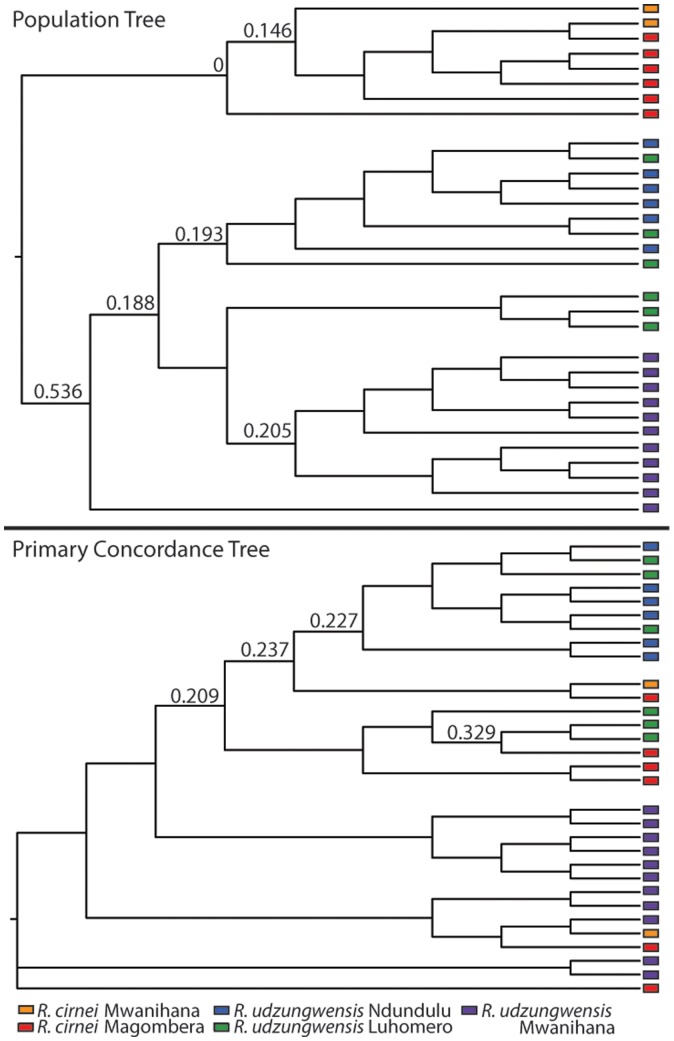
Bayesian concordance analysis in BUCKy: Population Tree (top), Primary concordance tree (bottom). Posterior mean concordance factors at major nodes are displayed above branches. Branches with concordance factors below 0.2 are not shown except in major nodes. The population tree shows monophyly for the two species, while the primary concordance tree shows admixture between the species and poorly supported relationships implying some level of admixture.

Species tree methods were in agreement concerning the monophyly of the species and phylogeographic relationships within species, with strong support (posterior = 1.00 for all three methods) for the basal split between the two species (Fig. 3). The BUCKy analysis, which was run with individuals as the terminals instead of populations as in the other species trees (e.g., [44]), showed monophyly in the Population Tree, yet extreme paraphyly in the Primary Concordance Tree (Fig. 4). As the CFs (concordance factors, which measure the genomic support of each clade) of the PCT were low, it is likely that many alternative topologies are possible for each node, consistent with Horizontal Gene Transfer.

### Species Distribution Models

#### Niche overlap between species

Geographic scale did not significantly impact the current distribution predictions or niche similarity estimates for these species, as models based on the full extent of Tanzania and models generated from just the rectangular region around the Udzungwa Mountains and Southern Highlands yielded similar results (Fig. S1). Projections into past climate conditions were less stable between the two scales, so results are interpreted from the limited distribution rectangle with insights from the larger scale. The AUC (Area Under the Curve) in the rectangle encompassing the Udzungwa Mountains was 0.901 for *R. c. reichardi*, and 0.994 for *R. udzungwensis*. The niches of the two species (*R. udzungwensis* and *R. c. reichardi*) are significantly more dissimilar than chance by all similarity measures (P<0.001; Fig. S2, S3). As expected given their separate distributions and elevation separation, all models confirmed that they currently occupy different niches despite their close spatial distribution.

#### Distributions and shifts

**Figure 5:**
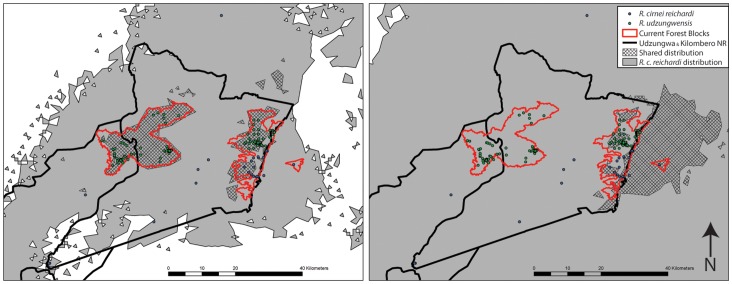
Species Distribution Models during current climate scenario (left) and wetter Last Interglacial (right; LIG). Current sampling points used in model construction are shown. The limited distribution of *R. udzungwensis* is entirely contained within the suitable area for *R. c. reichardi* at both time points. During the LIG, the ranges for both species were predicted to occur at lower elevations in the eastern areas of the Udzungwa Mountains foothills. During this time, *R. udzungwensis* shifted its entire distribution eastward resulting in a loss occupancy in the western Ndundulu/Luhomero forest, a reduced range in the Mwanihana forest, and a relatively extensive distribution east of Mwanihana in the Kilombero Valley including the Magombera forest (see Fig. 1 for place names).

Under current climate conditions, *R. c. reichardi* has a much larger and more continuous distribution in the region of interest than does *R. udzungwensis* (Fig. 5, left), which appears to be limited to the two extant forest blocks. The distributions during past wet climate cycles (exemplified by the LIG) show increased suitability throughout the region for *R. c. reichardi*. Instead of expanding in a similar manner to *R. c. reichardi*, *R. udzungwensis* appears to experience a shift in range towards eastern, lower-elevation forests. Instead of the current distribution with one population in a western high elevation forest (Ndundulu-Luhomero) and one population in a central mid-elevation forest (Mwanihana), the LIG distribution consists of populations in the central mid-elevation (Mwanihana) and the eastern lower-elevation forest of Magombera (currently only occupied by *R. c. reichardi*) (Fig. 5, right). The distribution of both species during dry climate cycles (exemplified by the LGM) was likely greatly reduced, as our climate models fail to predict any substantial areas of suitable habitat within this region for either species.

## Discussion

Species-tree methods confirm the genetic distinctness of the two species with very high support, validating the morphological description of *R. udzungwensis* [2]. This is a first, important result given the complex phylogeny of the genus. However, the discrepancy between the BUCKy population tree and primary concordance tree, and the clear introgression of mtDNA haplotypes despite nuclear monophyly imply that ancient hybridization occurred between these two taxa. The absence of current admixture inferred from monophyletic nuclear loci is supported by the fact that despite the close proximity of *R. c. reichardi* and *R. udzungwensis* populations in the Udzungwa Mountains, no morphologically intermediate individuals (presumed hybrids) have been found. Given the spatial proximity of these species, apparent mitochondrial introgression, and the narrow habitat requirements of *R. udzungwensis*, four related questions arise: (1) Why is there no evidence of nuclear gene introgression; (2) Why do some populations of *R. udzungwensis* exhibit ancient hybridization while others do not; (3) Why is there no evidence of current hybridization between the two species; and (4) Why do SDM models predict a near complete lack of suitable habitat in this area during the LGM?

### Mitochondrial Introgression

The phenomenon of extensive mitochondrial introgression without a comparable nuclear signal is relatively common in phylogenetic studies (e.g., [57]–[60], exemplified by a stark disagreement between resolved nuclear genes and admixed mitochondrial loci. Given the ∼4-fold increase in mutation rate for mtDNA vs. nuclear [61], [62], these patterns are unlikely to be a result of incomplete lineage sorting [63], particularly when two morphologically and genetically distinct lineages share exact mitochondrial haplotypes. Though data are extremely limited within this sengis, no examples of incomplete lineage sorting of mtDNA have been found [4], [64].

The signal of mitochondrial introgression can persist between species, despite loss of a nuclear signal when two potentially hybridizing species meet in circumstances of disparate abundance. In these uneven encounters, normally choosy females of the less abundant species may accept heterospecific mates if conspecifics mates are rare [58]. In these instances, though both nuclear and mitochondrial genes from the rare species introgress into the common species, mitochondrial introgression invariably exceeds nuclear. This is particularly apparent when the proportion of immigrants is small [58].

### Introgressed and Pure Populations

Historical uneven encounters could be expected in some populations of *R. udzungwensis* and *R. c reichardi*, while others may have remained isolated from introgression throughout history. Evidence from this study suggests that some populations have undergone introgression while others have not: (1) mtDNA of *R. udzungwensis* from the Luhomero forests appears introgressed into *R. c. reichardi* individuals from Magombera forest, (2) apparent mtDNA haplotypes from *R. c. reichardi* appear in *R. udzungwensis* individuals from Mwanihana, and (3) *R. udzungwensis* from Ndundulu appear isolated and free from admixture despite the fact that the Ndundulu forests are equally surrounded by *R. c. reichardi* as the other fragments occupied by *R. udzungwensis*.

The spatial distribution of habitat during both current and LIG climate conditions, and the unique nature of the Ndundulu forests offer possible explanations for spatially structured introgression. The Ndundulu forest appears to act as an ark of diversity for a number of forest-restricted species, providing stable habitat through time for its unusually divergent occupants (e.g., Udzungwa forest partridge (*Xenoperdix udzungwensis*, [65]–[67]), kipunji (*Rungwecebus kipunji,* [68], [69], and rufous-winged sunbird (*Cinnyris rufipennis*, [70]). The reason that distinct and relictual species are preserved in the Ndundulu forest is not understood, but perhaps various environmental aspects filter generalist species from invading and displacing or intermingling with local lineages.

Together, the current parapatric distribution, population-specific mtDNA introgression, and the predicted historical ranges of these species during interglacial cycles support a scenario of hybridization with *R. c. reichardi* through uneven encounters. As species ranges shifted in response to glacial climate cycles, rare colonists of displaced *R. udzungwensis* may have encountered abundant and established *R. c. reichardi* in the newly accessible flood plain forest of which Magombera is today a remnant (Fig. 5) leading to a transfer of mtDNA haplotypes into *R. c. reichardi* populations [71]. Likewise, *R. c. reichardi* may have expanded into the northern Mwanihana forests as *R. udzungwensis’* range contracted, leading to the mtDNA introgression from *R. c. reichardi* into *R. udzungwensis*. These scenarios of predicted relative abundance (i.e., “rare colonists” and “abundant established”) reflect stable range distributions of one species (based on current and LIG models) vs. newly occupied areas for the other. With the current data, much of this interpretation is speculative, but fits with known models of interactions. Future studies incorporating larger sampling of the diversity within *R. c. reichardi* and *R. cirnei* as a whole will help to clarify these hypotheses.

### Current Genetic Isolation

SDM show that the current *R. udzungwensis* range is completely within the predicted, and much broader, range of *R. c. reichardi*, yet no on-going hybridization appears to be taking place. In situations such as this, where one species is predicted to occupy the range of another yet is distinctly absent from that area, competition coupled with reinforcement for potentially hybridizing species is typically invoked. It is likely that the larger-bodied *R. udzungwensis* is able to outcompete *R. c. reichardi* at the higher/harsher elevations that it currently occupies, but *R. c. reichardi* is better able to utilize resources in the available landscape surrounding the *R. udzungwensis* refugia. In this case, gradual historic climate change may explain the trend towards reduced introgression at the present by reinforcing the concentration of *R. udzungwensis* in its wetter, highland refugia if it cannot compete in the drier mid-elevation areas occupied by *R. c. reichardi*. Though East Africa experienced many climate fluctuations in the past 5 my, oscillating from wet to dry climate conditions with associated forest expansions and contractions, there has been a general drying trend from 1.86 (+/−0.44 Ma) to present (ODP 721/722 dust flux record, [72]. From this point on, the range of *R. udzungwensis* would have concentrated in the highland forests of the Udzungwa Mountains tops instead of the expanded eastern range with *R. c. reichardi*. This scenario is difficult to verify without absolute dating of the larger phylogeny and the proposed introgression event (an ongoing effort of these authors and others), but is likely the levels of divergence and other phylogenetic and phylogeographic patterns in East Africa (discussed in [73]).

### SDM Model Predictions

Species distribution modelling is an invaluable tool for many aspects of evolutionary and conservation biology, but there are limitations for range-restricted species that tend to have little latitude in their environmental envelope [74]. The modelling approach in this study, MaxEnt based on bioclimatic variables, did not predict any areas of stable habitat (using a threshold cut-off of suitability) within the Udzungwa Mountains region for either sengi in this study during the LGM, a result that seems unlikely given our molecular findings. Though this may imply that populations in this area were nearly extinct, as should be investigated with a larger population genetics dataset, it is also possible that the distribution requirements of sengis are either not adequately predicted by these models or that the reconstructions of climate during the LGM may be inadequate for this region. Future improvements in both models and sengi distribution requirements will aid in our understanding of these dynamics: (1) Models of past and future climate continue to evolve, particularly in terms of sensitivity to the under-represented African tropics. These advancements will greatly enhance the understanding of climatic effects on diversity within African lineages as a whole. (2) As more ecological information becomes available for sengis including microhabitat requirements, physiological limits, and potential competitors, we may be able to provide a more sensitive model of distributions. These traits, however, have limited ability to be predicted into past and future climates.

### Advancing our Understanding of Sengis

This study is the first known case of introgression in sengis, a result that was only identifiable using multi-locus data from multiple populations. Previous investigations into relationships of co-distributed sengis failed to detect introgression, but lacked multi-locus data [4], [8]. A multi-locus perspective will be necessary in studies of sengis, including phylogenetic analyses, to adequately interpret interspecific relationships.

In the current study, we are able to see a clear signal of introgression using multiple individuals from multiple localities. In future studies, increased sample sizes sufficient to investigate demographic events (such as expansions and contractions) and estimate population diversity across ranges would both verify proposed demographic processes and ground our estimates of the extent of introgression between species.

This study demonstrates that multi-locus, population-level sampling including introgression analyses is imperative for any attempts at investigating the broader relationships and evolutionary history of *Rhynchocyon*. Within this, all subspecies, pelage variants, and allopatric populations should be considered for the fullest understanding of evolution and diversification within this poorly understood genus. It also highlights how comparisons with neighboring and closely related species highlight the uniqueness and complex history of a new and vulnerable giant sengi.

## Supporting Information

Figure S1 ENM predictions of Tanzania vs. reduced rectangle. Current predictions for *R. c. reichardi* (left) and *R. udzungwensis* (right) with Udzungwa NP and Kilombero Forest reserve outlined in black for reference.
(TIF)

Figure S2 Niche Identity comparisons between *R. udzungwensis* and *R. c. reichardi.*
(TIF)

Figure S3 Similarity of *R. udzungwensis* and *R. c. reichardi* niche to backgrounds. Top of each set: Shared background of entire evaluated rectangle. Bottom of each set: compared to random points in the threshold occurrence envelope of the other species. Arrows represent actual similarity.
(TIF)

Table S1 Collection and identification of specimens used in this study.
(XLS)

Text S1 Supplementary information regarding jModelTest, BPP, exclusion of 12S numts, niche comparisons between *R. udzungwensis* and *R. c. reichardi.*
(DOCX)
