# Supplementary figures and images for: Evolutionary History of the Grey-Faced Sengi, Rhynchocyon udzungwensis, from Tanzania: A Molecular and Species Distribution Modelling Approach
Source: PLoS One. 2013 Aug 27;8(8):e72506. doi: 10.1371/journal.pone.0072506 (PMC3754996; doi:10.1371/journal.pone.0072506)

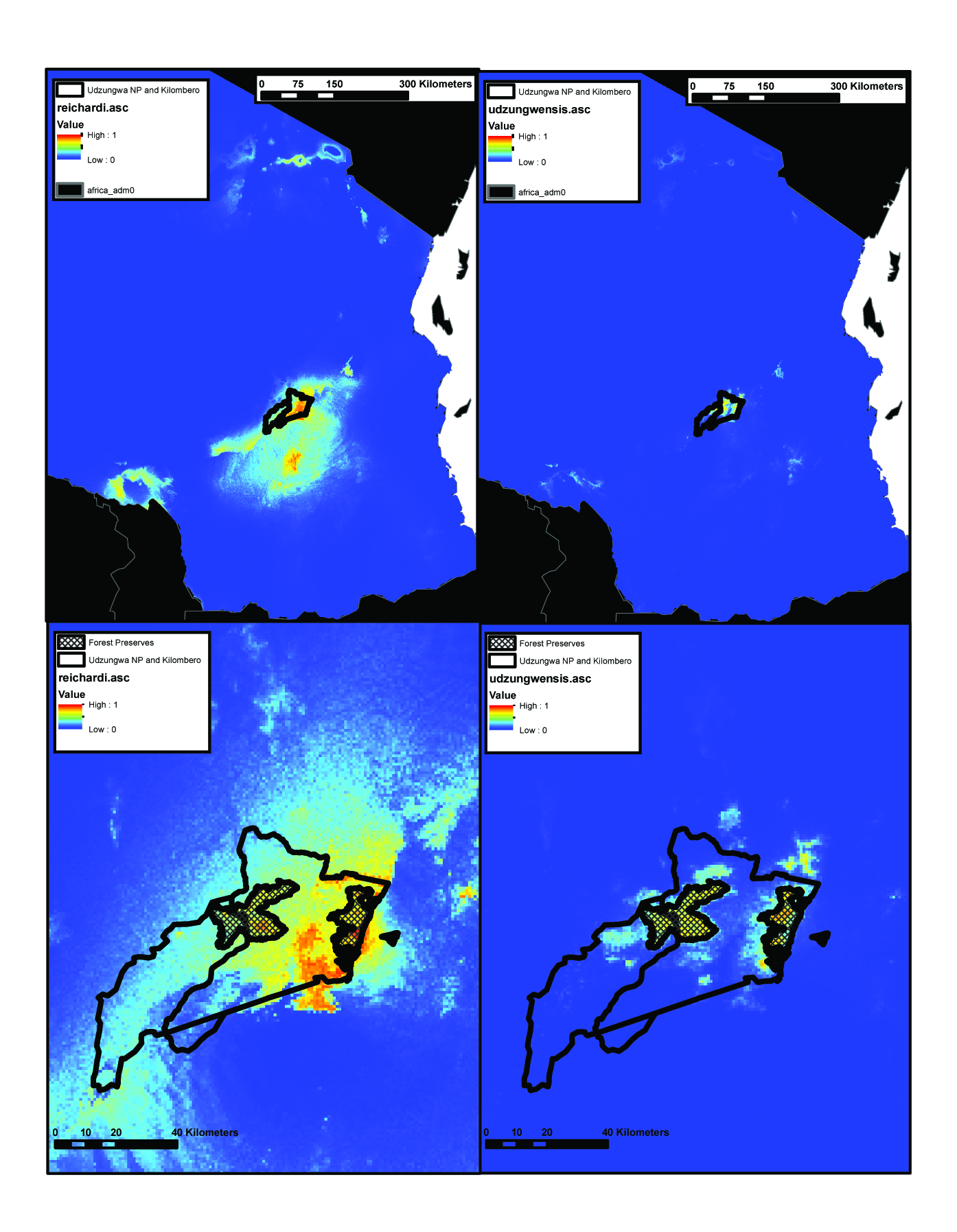

Supplement: Figure S1 — ENM predictions of Tanzania vs. reduced rectangle. Current predictions for R. c. reichardi (left) and R. udzungwensis (right) with Udzungwa NP and Kilombero Forest reserve outlined in black for reference. (TIF) [file pone.0072506.s001.tif]

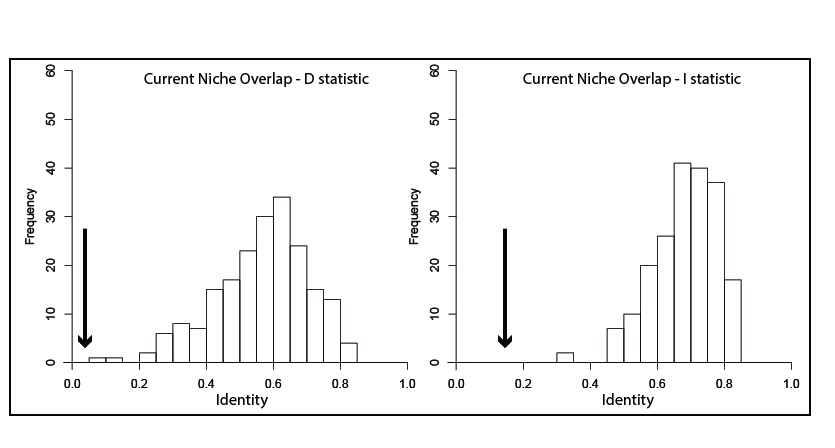

Supplement: Figure S2 — Niche Identity comparisons between R. udzungwensis and R. c. reichardi. (TIF) [file pone.0072506.s002.tif]

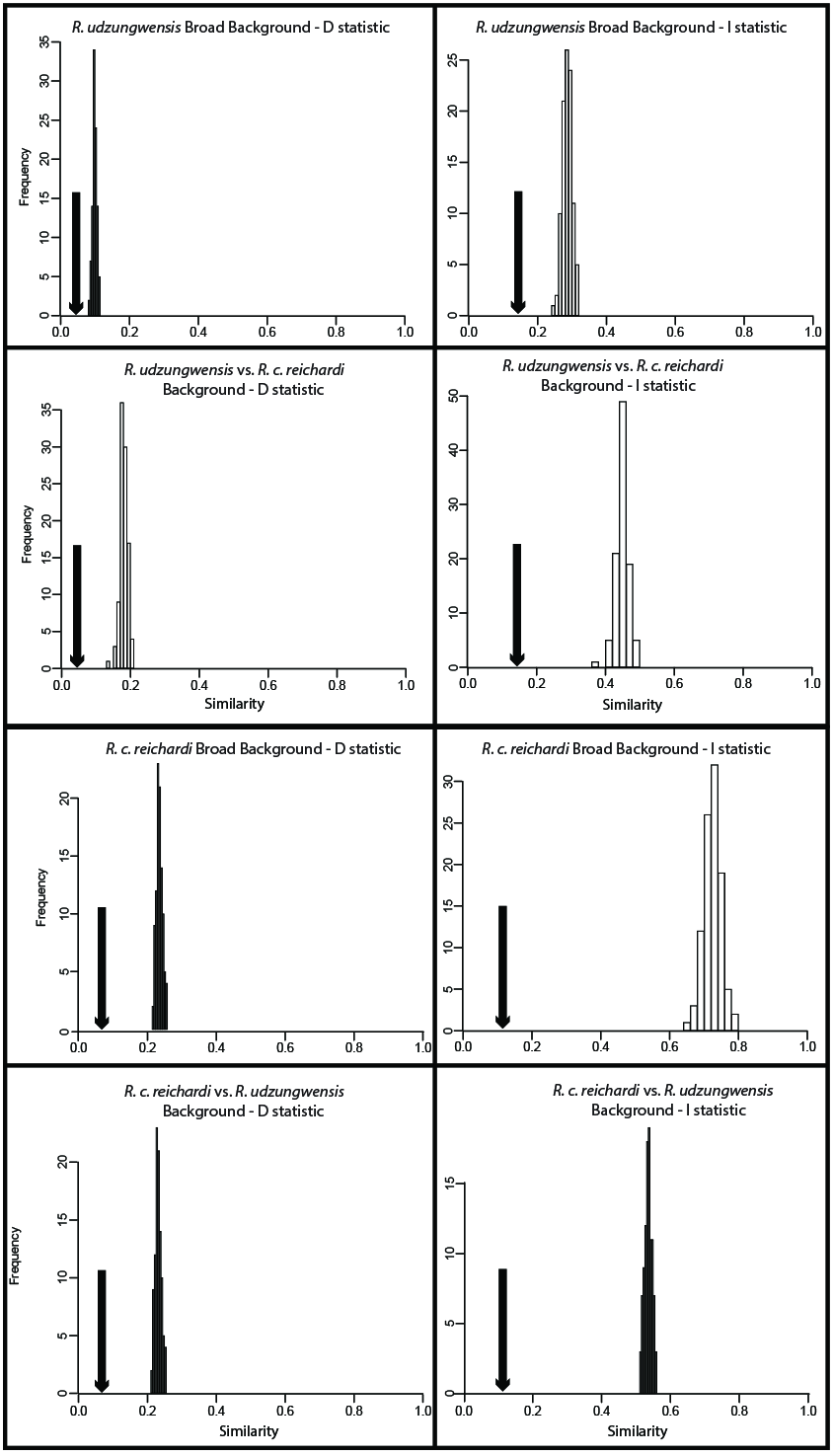

Supplement: Figure S3 — Similarity of R. udzungwensis and R. c. reichardi niche to backgrounds. Top of each set: Shared background of entire evaluated rectangle. Bottom of each set: compared to random points in the threshold occurrence envelope of the other species. Arrows represent actual similarity. (TIF) [file pone.0072506.s003.tif]
