# Supplementary material for: Evolutionary History of the Grey-Faced Sengi, Rhynchocyon udzungwensis, from Tanzania: A Molecular and Species Distribution Modelling Approach
Source: PLoS One. 2013 Aug 27;8(8):e72506. doi: 10.1371/journal.pone.0072506 (PMC3754996; doi:10.1371/journal.pone.0072506)
Supplement: Text S1 — Supplementary information regarding jModelTest, BPP, exclusion of 12S numts, niche comparisons between R. udzungwensis and R. c. reichardi. (DOCX) [file pone.0072506.s005.docx]

Supplementary Materials

**Jmodeltest** – top 10 models based on AICc values. Top model represented in BEST selected.

| vWFB |  |  |  |
| --- | --- | --- | --- |
| Model | -lnL | AICc | delta |
| TPM2uf | 1608.0608 | 3358.951 | 0 |
| TPM1uf | 1608.1913 | 3359.2121 | 0.2611 |
| TPM3uf | 1608.3902 | 3359.6098 | 0.6588 |
| HKY | 1609.586 | 3359.7334 | 0.7824 |
| TIM2 | 1607.9038 | 3360.9094 | 1.9583 |
| TVM | 1606.8648 | 3361.1082 | 2.1571 |
| TIM1 | 1608.0346 | 3361.1709 | 2.2199 |
| TPM2 | 1612.5839 | 3361.2065 | 2.2555 |
| TPM2uf+G | 1608.0633 | 3361.2285 | 2.2774 |
| TPM2uf+I | 1608.1078 | 3361.3174 | 2.3663 |
|  |  |  |  |
| ENAM |  |  |  |
| Model | -lnL | AICc | delta |
| TPM2uf+I | 3672.0311 | 7483.7728 | 0 |
| HKY+I | 3673.3423 | 7484.2862 | 0.5134 |
| TIM2+I | 3671.6868 | 7485.1947 | 1.4219 |
| TrN+I | 3672.9863 | 7485.6832 | 1.9104 |
| TPM2uf+G | 3673.062 | 7485.8345 | 2.0617 |
| TPM2uf+I+G | 3672.0298 | 7485.8808 | 2.1079 |
| TPM2uf | 3674.2356 | 7486.0727 | 2.2999 |
| TPM1uf+I | 3673.1919 | 7486.0943 | 2.3215 |
| TPM3uf+I | 3673.2251 | 7486.1607 | 2.3879 |
| HKY+G | 3674.3459 | 7486.2934 | 2.5206 |
|  |  |  |  |
| Combined mtDNA | |  |  |
| Model | -lnL | AICc | delta |
| HKY+I | 3113.5651 | 6366.5283 | 0 |
| TPM3uf+I | 3112.8842 | 6367.3309 | 0.8025 |
| TPM1uf+I | 3113.2461 | 6368.0548 | 1.5265 |
| TrN+I | 3113.3632 | 6368.2889 | 1.7606 |
| TPM2uf+I | 3113.4462 | 6368.4549 | 1.9266 |
| HKY+I+G | 3113.5627 | 6368.688 | 2.1596 |
| TIM3+I | 3112.6929 | 6369.1153 | 2.587 |
| TIM1+I | 3113.036 | 6369.8015 | 3.2731 |
| TIM2+I | 3113.2076 | 6370.1447 | 3.6163 |
| TrN+I+G | 3113.3622 | 6370.454 | 3.9256 |

**BPP alternative**

A0, e20

((RciMago, RciMwah)'#0.45487', ((RudUdz, RudLuho)'#0.26419', (RudMwan, RudMwaS)'#0.28338')'#1.00000')'#1.00000';

1 1 2 1

((RciMago, RciMwah)'#0.44319', ((RudUdz, RudLuho)'#0.24781', (RudMwan, RudMwaS)'#0.25360')'#1.00000')'#1.00000';

**Exclusion of 12S numts.**

We tested, in two randomly chosen individuals, whether the sequence of about 650 bp obtained with the original primer pairs described in Material and Methods was the same as that obtained by sequencing several clones of a longer PCR product, amplified with a different primer pair. It is considered very unlikely to obtain the same NuMt with independent PCR primers [1], especially when the PCR products differ in length.

The following primers were used for amplifying about 1 kb of the 12S marker: F-5'-AAAGCAAAAGCACTGAAAATG-3' and R 5' TGACTGCAGAGGGTGACGGGCGGTGTGT-3'. The thermal profile was as follows: incubation for 2 min at 94 °C, 40 cycles of denaturation at 94 °C (30 s), annealing at 50 °C (30 s) and extension at 70 °C (1 min and 30 s) followed by a final extension step of 7 min at 70 °C. Cloning of PCR products was accomplished with TOPO TA commercial kit (Invitrogen, UK), following manufacturer's instructions. At least ten different clones per individual were sequenced with M13 primers. Only a few singletons of difference were observed among the different clones, compatible with the standard *Taq* polymerase error rate of 7.3 x 10^-5^ per bp per duplication [2]. The final length of the consensus sequence was 800 bp. The manual alignment of this 800 bp sequence with the shorter 650 bp one revealed a 100 % homology. Considering also the high amount and quality of our starting DNA, extracted from muscle tissue, we therefore concluded that occurrence of NuMts can be confidently excluded.

**Table of Actual, Identiy, and background comparisons** for both D and I statistics on niche identity between *R. c. reichardi* and *R. udzungwesis.*

| **D statistic** |  |  |  | **I statistics** |  |  |
| --- | --- | --- | --- | --- | --- | --- |
|  | mean | SD |  |  | mean | SD |
| Actual | 0.0321 |  |  | Actual | 0.1791 |  |
| Identity | 0.5648 | 0.1413 |  | Identity | 0.6827 | 0.0964 |
| *R. c. reichardi* to *R. udzungwesis* Background | 0.2315 | 0.0093 |  | *R. c. reichardi* to *R. udzungwesis* Background | 0.5353 | 0.0107 |
| *R. udzungwesis* to *R. c. reichardi* Background | 0.1808 | 0.0118 |  | *R. udzungwesis* to *R. c. reichardi* Background | 0.4488 | 0.0186 |
| *R. udzungwesis* to shared Background | 0.0992 | 0.0063 |  | *R. udzungwesis* to shared Background | 0.2860 | 0.0139 |
| *R. c. reichardi* to shared Background | 0.4602 | 0.0255 |  | *R. c. reichardi* to shared Background | 0.7243 | 0.0248 |

***References***

1. Bensasson D, Zhang D-X, Hartl DL, Hewitt GM (2001) Mitochondrial pseudogenes: evolution’s misplaced witnesses. Trends Ecol Evol 16: 314–321.

2. Kobayashi N, Tamura K, Aotsuka T (1999) PCR Error and Molecular Population Genetics. Biochem Genet 37: 317–321. doi:10.1023/A:1018759210666.
